# Space-time clustering analyses of type 1 diabetes in children from north-east England: support for an infectious aetiology?

**DOI:** 10.1186/1476-069X-8-S1-S14

**Published:** 2009-12-21

**Authors:** Richard JQ McNally, Raymond Pollock, Simon Court, Mike Begon, Tim D Cheetham

**Affiliations:** 1 Institute of Health and Society, Newcastle University, UK; 2 Institute of Human Genetics, Newcastle University, UK; 3 School of Biological Sciences, Liverpool University, UK

## Abstract

**Background:**

The aetiology of type 1 diabetes in children is uncertain. A number of recent studies have suggested an infectious aetiology. It has been postulated that an infectious agent may be involved. Support for this hypothesis may be provided by a finding of space-time clustering. The aims of this study were: (i) to determine whether there was space-time clustering in cases of childhood diabetes from north-east England; and to test for differences in space-time clustering: (ii) due to age at diagnosis; (iii) between the sexes and (iv) between levels of residential population density.

**Methods:**

We studied incidence of type 1 diabetes diagnosed in children aged 0-14 years and diagnosed during the period 1990-2007. All cases were resident in a defined geographical region of north-east England (Northumberland, Newcastle upon Tyne and North Tyneside). We applied a second-order procedure based on *K*-functions to test for global clustering. Locations were residential addresses at time of diagnosis. Tests were repeated using nearest neighbour thresholds to allow for variable population density, providing the primary result for each analysis. Differences between sexes and between levels of population density were assessed.

**Results:**

We analysed 457 cases of type 1 diabetes. Overall, there was marginally significant evidence of global space-time clustering (*P *= 0.089). There was statistically significant clustering amongst pairs of cases that contained at least one female (*P *= 0.017), but not amongst pairs of cases that contained at least one male (*P *= 0.190). Furthermore, there was significant clustering amongst pairs of cases that contained at least one from a more densely populated area (*P *= 0.044), but not amongst pairs of cases that contained at least one from a less densely populated area (*P *= 0.226).

**Conclusion:**

Although the analyses have only found marginally significant evidence of global space-time clustering for cases of type 1 diabetes diagnosed in north-east England, there were two notable findings. First, there was evidence of clustering amongst females and secondly clustering was confined to cases from more densely populated areas. These findings are consistent with a possible aetiological involvement of an infectious agent.

## Introduction

The aetiology of type 1 diabetes is uncertain, although a number of studies have suggested a role for infections and immunological responses. Higher incidence has been associated with better hygiene and later exposure to infections[1-3]. Viruses that have been postulated include enterovirsues, rotavirus, mupms, cytomegalovirus and rubella[4]. Recent studies have also suggested that Ljungan virus (a picornavirus) may be involved in the causation of type 1 diabetes in rodents [5,6]. It is possible that it may act similarly in human populations [7].

Space-time clustering is observed when excess numbers of cases are observed within small geographical locations for limited periods of time, and these excesses cannot be explained in terms of general excesses in those locations or at those times [8]. A finding of space-time clustering is consistent with the role of an environmental factor in aetiology, such as an infection.

A previous study, from Yorkshire, UK, has found evidence of space-time clustering in 10-14 and 15-19-year olds [9]. However, it is not clear whether space-time clustering is restricted to that geographical region, or whether such patterning is present elsewhere. To address this question, we have analysed space-time clustering in children (aged 0-14 years) who were diagnosed with type 1 diabetes in a geographically-defined region of north-east England.

The aims of the present study were: (i) to determine whether there was space-time clustering in cases of childhood diabetes from north-east England; and to test for differences in space-time clustering: (ii) due to age at diagnosis; (iii) between the sexes; and (iv) between levels of residential population density.

## Methods

The study analysed children (aged 0-14 years) who were diagnosed with type 1 diabetes during the period 1^st ^January 1990 - 31^st ^December 2007 and resident in a geographically defined region of north-east England (Northumberland, Newcastle upon Tyne and North Tyneside). The area has a stable population, with low levels of inward or outward migration [10,11]. Furthermore, the population is ethnically homogeneous, with fewer than 2% from ethnic minorities [12]. Cases were identified from several independent sources (paediatric and adult clinical data bases, admission diaries, in/out patient records), thus ensuring that there was a high level of ascertainment. There were no known temporal or geographical biases.

Ordnance Survey four-digit grid references (Easting and Northing) were allocated to each case with respect to the centroid of the postcode of the residential address at diagnosis, locating each address to the nearest 0.1 km. In the whole of the UK there are approximately 1.7 million postcodes. These uniquely identify addresses for postal delivery. A single postcode may identify fifteen to twenty houses, a smaller number of multiple occupancy residences, or a single commercial building [13]. The space-time analyses were based on time and place of diagnosis.

The following hypotheses were tested: (i) there is spatio-temporal heterogeneity in the incidence of type 1 diabetes in children residing in north-east England; (ii) geographical or temporal heterogeneity of incidence of type 1 diabetes is modulated by age; (iii) geographical or temporal heterogeneity of incidence of type 1 diabetes is modulated by sex; and (iv) geographical or temporal heterogeneity of incidence of type 1 diabetes is modulated by differences in patterns of exposure related to level of population density.

We analysed global space-time clustering using a method based on *K*-functions [14]. This method may be regarded as being a generalisation of the Knox test [15]. Briefly, in the Knox test a pair of cases is said to occur in "close proximity" if both dates of diagnosis and residential addresses at time of diagnosis are close. The number of pairs of cases observed to be in close proximity (denoted *O*) is obtained and the number of pairs of cases expected to be in close proximity is calculated (denoted *E*). If *O *is greater than *E*, then there is evidence of space-time clustering.

There is an underlying problem with the Knox test. Boundaries chosen to define "close proximity" in time and space are entirely arbitrary. Repeating of the Knox test, using a number of different boundaries in time and space, leads to multiple testing. A simplified second-order procedure, based on *K*-functions, is used to partly deal with this problem [14]. In this method, a set of Knox-like calculations are performed, where the critical values change over a pre-specified grid. For close times, we have chosen *t *= 0.1, 0.2,....., 1.5 years and for close distances we have chosen *s *= 0.5, 1, 1.5,....., 7.5 km.

The method of analysis based on fixed geographical distances makes no allowances for underlying heterogeneity in population density. North-east England has a highly variable population distribution, since it includes both urban and rural areas. Any specified distance between two cases may have different meanings in urban and rural areas. For example the size of school catchment areas will be different. Use of fixed geographical distance thresholds may lead to an underestimation of expected numbers in more densely populated areas, leading to a possible inflation of the clustering effect. Conversely, the use of fixed geographical thresholds may lead to an overestimation of expected numbers in less densely populated areas, leading to a possible deflation of the clustering effect.

To allow for variations in population density we repeated the analyses, replacing fixed geographical distances by variable nearest neighbour (NN) distances to the (*N*-7)^th^,....., (*N*+7)^th ^NN's, using the diagnosis locations of all the cases in the data set. *N *was chosen such that the mean distance was 5 km and was found to be *N *= 21. This method provided the primary result for each analysis and is similar to a method originally proposed by Jacquez [16].

We analysed the following sub-age-groups: 0-4 years, 5-9 years and 10-14 years. We analysed sex by considering: (i) clustering pairs that included at least one male case (i.e. "male: any" pairs) and (ii) clustering pairs that included at least one female case (i.e. "female: any" pairs).

Cases were assigned to a "more densely populated" group if the 21^st ^NN was nearer than the median distance (2.5 km) of all the 21^st ^NNs and to a "less densely populated" group otherwise. Analysis by the two levels of population density was carried out for clustering pairs that included at least one case from the "more densely populated" group (i.e. "more densely populated: any" pairs) and also for clustering pairs that included at least one case from the "less densely populated" group (i.e. "less densely populated: any" pairs). It should be noted that analyses of population density may be diluted because the areas (especially "less densely populated") are not contiguous.

The *K*-function analyses were performed using 999 simulations. Statistical significance was indicated if *P *< 0.05 and marginal significance if 0.05 ≤ *P *< 0.10.

## Results

We analysed 457 cases of type 1 diabetes, comprising 227 males, 230 females, 115 aged 0-4 years, 156 aged 5-9 years and 186 aged 10-14 years. Overall, there was marginally significant evidence of global space-time clustering (*P *= 0.240 using the fixed geographical distance version of the *K*-function method and *P *= 0.089 using the variable NN threshold version of the *K*-function method) [Table 1].

**Table 1:** Results of *K*-function analyses of space-time clustering for cases of type 1 diabetes diagnosed in north-east England during the period 1990-2007^a,b^

Group	Geographical distance^c^	NN threshold^d^
All case pairs	*P *= 0.240	*P *= 0.089
Case pairs aged 0-4 years only	*P *= 0.343	*P *= 0.541
Case pairs aged 5-9 years only	*P *= 0.112	*P *= 0.187
Case pairs aged 10-14 years only	*P *= 0.318	*P *= 0.478
"Male: any" case pairs	*P *= 0.453	*P *= 0.190
"Female: any" case pairs	*P *= 0.182	*P *= 0.017
"More densely populated: any" case pairs	*P *= 0.211	*P *= 0.044
"Less densely populated: any" case pairs	*P *= 0.350	*P *= 0.226

^a^Cases are temporally close if dates of diagnosis differ by <*t *where *t *varies from 0.1 year to 1.5 years in steps of 0.1 year;

^b^*P*-value obtained by simulation (999 runs) with dates of diagnosis randomly re-allocated to the cases in the analysis;

^c^Cases are spatially close if locations at diagnosis differ by <*s *where *s *varies from 0.5 km to 7.5 km in steps of 0.5 km;

^d^Cases are spatially close if either is within the distance to the *N*^th ^NN of the other where *N *varies from 14 to 28 in steps of 1.

There was no evidence of clustering in any sub-age-group (ages 0-4: *P *= 0.343 and *P*= 0.541 using the fixed geographical distance and variable NN threshold versions of the *K*-function method, respectively; ages 5-9: *P*= 0.112 and *P *= 0.187; ages 10-14: *P *= 0.318 and *P *= 0.478) [Table 1].

There was statistically significant space-time clustering amongst "female: any" case pairs (*P *= 0.182 and *P *= 0.017 using the fixed geographical distance and variable NN threshold versions of the *K*-function method, respectively). However, there was no evidence of clustering amongst "male: any" case pairs (*P *= 0.453 and *P *= 0.190) [Table 1].

Also, there was statistically significant space-time clustering amongst "more densely populated: any" case pairs only (*P *= 0.211 and *P *= 0.044 using the fixed geographical distance and variable NN threshold versions of the *K*-function method, respectively). Clustering was not significant amongst "less densely populated: any" case pairs (*P *= 0.350 and *P *= 0.226) [Table 1].

## Discussion

Although the analyses have only found marginally significant evidence of global space-time clustering for cases of type 1 diabetes diagnosed in north-east England, there were two notable findings. First, there was evidence of clustering amongst females and secondly clustering was confined to cases from more densely populated areas. These findings suggest the possible aetiological involvement of an infectious agent. It should be noted that heterogeneity between- individuals in the latency from initial infection until onset of type 1 diabetes is likely to have diluted the overall clustering effect.

The results are consistent with findings of space-time clustering from Yorkshire. The analyses of type 1 diabetes from that area found space-time clustering in 10-19 year olds. The clustering amongst 10-14 year olds was also more marked in case pairs that included at least one female and in case pairs that included at least one from a more densely populated area [9].The findings of clustering amongst females is consistent with earlier onset of puberty and differential pubertal effects for males and females on the immune system [17], or differences in patterns of exposure between the sexes (possibly via social contact).

Our new analyses from north-east England confirm the possibility of an infectious aetiology. The findings also indicate possible sex-related differences in response to an infectious agent. Furthermore the putative agent may be more prevalent in more urban locations.

Further research will examine geographical heterogeneity of incidence of type 1 diabetes in north-east England and will seek to link any patterning with prevalence of putative aetiological agents (such as Ljungan virus). Additional funding is currently being sought.

## List of abbreviations used

*O*: number of cases observed to be in close proximity; *E*: number of cases expected to be in close proximity; NN: nearest neighbour.

## Note

The peer review of this article can be found in Additional file 1.

## Competing interests

The authors declare that they have no competing interests.

## Authors' contributions

RJQMcN analysed the data and wrote the first version of the manuscript. RP, SC and TDC were involved in the process of data collection. All authors contributed critically to the study design, data analysis and interpretation and approved the final version of the manuscript.

## Supplementary Material

Additional file 1 Peer review Click here for file
