# Supplementary material for: Space-time clustering analyses of type 1 diabetes in children from north-east England: support for an infectious aetiology?
Source: Environ Health. 2009 Dec 21;8(Suppl 1):S14. doi: 10.1186/1476-069X-8-S1-S14 (PMC2796492; doi:10.1186/1476-069X-8-S1-S14)
Supplement: Additional file 1 — Peer review [file 1476-069X-8-S1-S14-S1.doc]

| Article ref no. | A1471-2105X SZ-S13 |
| --- | --- |
| Title | **Space-time clustering analyses of type 1 diabetes in children from north-east England.** |
| Author(s) | McNally, R.J.Q., Pollock, R., Court, S., Begon, M. and Cheetham, T.D. |

**Ref#1**

**General comments:**

The study was conducted to determine whether support for an infectious etiology of type 1 diabetes could be found by evidence of space-time clustering of cases in a defined geographical area. It is stated that the findings suggest that there is support for the hypothesis; however, some more explanation and discussion is necessary to make this suggestion clear.

**Major compulsory revisions:**

None

Minor essential revisions:

###### Title

The title does not fully reflect the aims of the work. Suggest something like: Space-time clustering analyses of type 1 diabetes in children from north-east England: support for an infectious etiology?

- **AUTHOR RESPONSE: Title changed as suggested [Title]**

###### Abstract

The involvement of Ljungan virus in diabetes is only a possibility, so it is better if it is not mentioned explicitly in the abstract. Suggest changing 3^rd^ sentence to “It has been postulated that an infectious agent may be involved”.

- **AUTHOR RESPONSE: Wording changed as suggested [Abstract, Background, third sentence]**

Sentence 4 states that the hypothesis would be supported if space-time clustering is found. It therefore needs to be briefly stated in the abstract why tests ii, iii and iv were performed.

- **AUTHOR RESPONSE: Tests ii, iii, iv refer to differences in space-time clustering. The words “in space-time clustering” have been added both here & in the last paragraph of the Introduction.**

###### Introduction

1^st^ para: change “plausible” to “possible”.

- **AUTHOR RESPONSE: Wording changed [Introduction, first paragraph, final sentence]**

###### Discussion

I can understand why case clustering in areas of high population density is suggestive of an etiological involvement, but why is clustering amongst females likewise suggestive? This should be clearly explained.

- **AUTHOR RESPONSE: The finding of clustering amongst females is consistent with earlier onset of puberty and differential pubertal effects for males and females on the immune system, or differences in patterns of exposure between the sexes (possibly via social contact). Explanation given & reference added [Discussion, paragraph 2 final sentence & new reference 17].**

“Further research will…” . Is this research currently being performed? If so, could some brief details be provided (e.g. name of project, funding body)? This would be helpful to those interested in following the progress of this topic.

- **AUTHOR RESPONSE: Additional funding is currently being sought [Discussion, final sentence].**

**Ref#2**

**General comments:** This is an interesting and potentially important study which deals with an area of ongoing controversy; namely the possible involvement of an infectious agent in type 1 diabetes. The methodology is explained carefully and appears to have been applied appropriately. The results achieve marginal significance and the authors explain these in the context of other relevant work.

**Major compulsory revisions:**

An important issue that is relevant to a study of this type, is the time interval between possible infection with a virus and the onset of disease. For example, it has not been established that any two individuals will progress to diabetes with a similar time course even if the underlying disease pathology is initiated concordantly. Thus, it seems probable that a common viral infection at one time point may lead to disease outcomes at a very different time point among different individuals (which may explain, in part, the lack of disease concordance in identical twins). Such divergence seems important to the interpretation of the present study and should be given some consideration in the Discussion section.

- **AUTHOR RESPONSE: We recognize this important point. We have added the following sentence – “It should be noted that heterogeneity between- individuals in the latency from initial infection until onset of type 1 diabetes is likely to have diluted the overall clustering effect” [Discussion, first paragraph, final sentence].**

**Minor Essential revisions:**

Lundgren virus is cited as a possible infectious agent associated with type 1 diabetes but there are a range of additional viruses which seem much more likely candidates (see e.g. Filippi; Diabetes 57, 2863, 2008.). Thus, the Introduction could be improved by inclusion of references to these additional agents.

- **AUTHOR RESPONSE: We have added a sentence and a reference to acknowledge that viruses that have been postulated include enteroviruses, rotaviruses, mumps, cytomegalovirus and rubella. We have also stated that higher incidence has been associated with better hygiene and later exposure to infections [Introduction, first paragraph, second and third sentences].**
